# Supplementary material for: Micronutrient supplements can promote disruptive protozoan and fungal communities in the developing infant gut
Source: Nat Commun. 2021 Nov 18;12:6729. doi: 10.1038/s41467-021-27010-3 (PMC8602372; doi:10.1038/s41467-021-27010-3)
Supplement: Supplementary file 1 — Supplementary Information [file 41467_2021_27010_MOESM1_ESM.pdf]

Supplementary Information

Micronutrient supplements can promote disruptive protozoan and fungal communities in the developing infant gut

Ana Popovic[1,2], Celine Bourdon[3,4], Pauline W. Wang[5,6], David S. Guttman[5,6], Sajid Soofi[7], Zulfiqar A. Bhutta[4,7], Robert H. J. Bandsma[3,4], John Parkinson[1,2,8,*] and Lisa G. Pell[4]

[1]Program in Molecular Medicine, Hospital for Sick Children Research Institute
[2]Department of Biochemistry, University of Toronto, Toronto, Ontario, Canada
[3]Division of Gastroenterology, Hepatology and Nutrition, Hospital for Sick Children, Toronto, Ontario, Canada
[4]Centre for Global Child Health, Hospital for Sick Children, Toronto, Ontario, Canada
[5]Department of Cell & Systems Biology, University of Toronto, Toronto, Ontario, Canada
[6]Centre for the Analysis of Genome Evolution & Function, University of Toronto, Toronto, Ontario, Canada
[7]Center of Excellence in Women and Child Health, the Aga Khan University, Karachi, Pakistan
[8]Department of Molecular Genetics, University of Toronto, Toronto, Ontario, Canada

Supplementary Table 1. Participant characteristics in the parent cRCT (ClinicalTrials.gov identifier NCT00705445) and microbiome substudy.

**Parent cRCT (NCT00705445)**	**Control**	**MNP**	**MNP with zinc**
Participants followed to 24 mo, n(%)	671 (34.0%)	646 (32.7%)	659 (33.4%)
Rural site, n(%)	402 (59.9%)	409 (63.3%)	415 (63.0%)
Female, n(%)	334 (49.8%)	323 (50.0%)	322 (48.9%)
Premature birth, n(%)	190 (28.3%)	151 (23.4%)	204 (31.0%)
Initiated breastfeeding, n (%)	663 (98.8%)	639 (98.9%)	659 (100.0%)
Breastfeeding status at 6 mo, n(%)			
Exclusive	24 (3.6%)	16 (2.5%)	14 (2.1%)
Partial	617 (92.1%)	603 (93.3%)	623 (94.5%)
None	29 (4.3%)	27 (4.2%)	22 (3.3%)
Anthropometry, 24 mo			
Weight, Kg	9.6 (9.5 - 9.6)	9.5 (9.4 - 9.6)	9.5 (9.4 - 9.6)
Length, cm	79.8 (79.5 - 80.1)	79.5 (79.1 - 79.8)	79.7 (79.4 - 80.0)
Weight-for-length, z-score	-0.9 (-1.0 - -0.9)	-0.9 (-1.0 - -0.9)	-1.0 (-1.1 - -0.9)

**Microbiome substudy**	**Control**	**MNP**	**MNP with zinc**
Participants, n(%)	24 (30.0%)	29 (36.2%)	27 (33.7%)
Rural site, n(%)	18 (75%)	19 (65.5%)	16 (59.3%)
Female, n(%)	12 (50%)	18 (62.1%)	14 (51.9%)
Premature birth, n(%)	5 (20.8%)	7 (24.1%)	4 (14.8%)
Initiated breastfeeding, n (%)	22 (91.7%)	29 (100%)	27 (100%)
Breastfeeding status at 6 mo, n(%)			
Exclusive	0 (0%)	0 (0%)	0 (0%)
Partial	23 (95.8%)	28 (96.6%)	27 (100%)
None	1 (4.2%)	1 (3.4%)	0 (0%)
Anthropometry, 24 mo			
Weight, Kg	9.3 (8.8 - 9.9)	9.3 (8.7 - 10)	9.9 (9.3 - 10.6)
Length, cm	78.9 (77.9 - 80)	80.1 (78.5 - 81.8)	80.3 (78.9 - 81.8)
Weight-for-length, z-score	-1.1 (-1.7 - -0.4)	-1.3 (-2 - -0.7)	-0.7 (-1.4 - 0)

Categorical values are presented as n (%), continuous variables show the mean and 95% confidence intervals.

Premature birth was defined as gestational age < 37 months.

Initiation of breastfeeding was reported for the period prior to recruitment into the study.

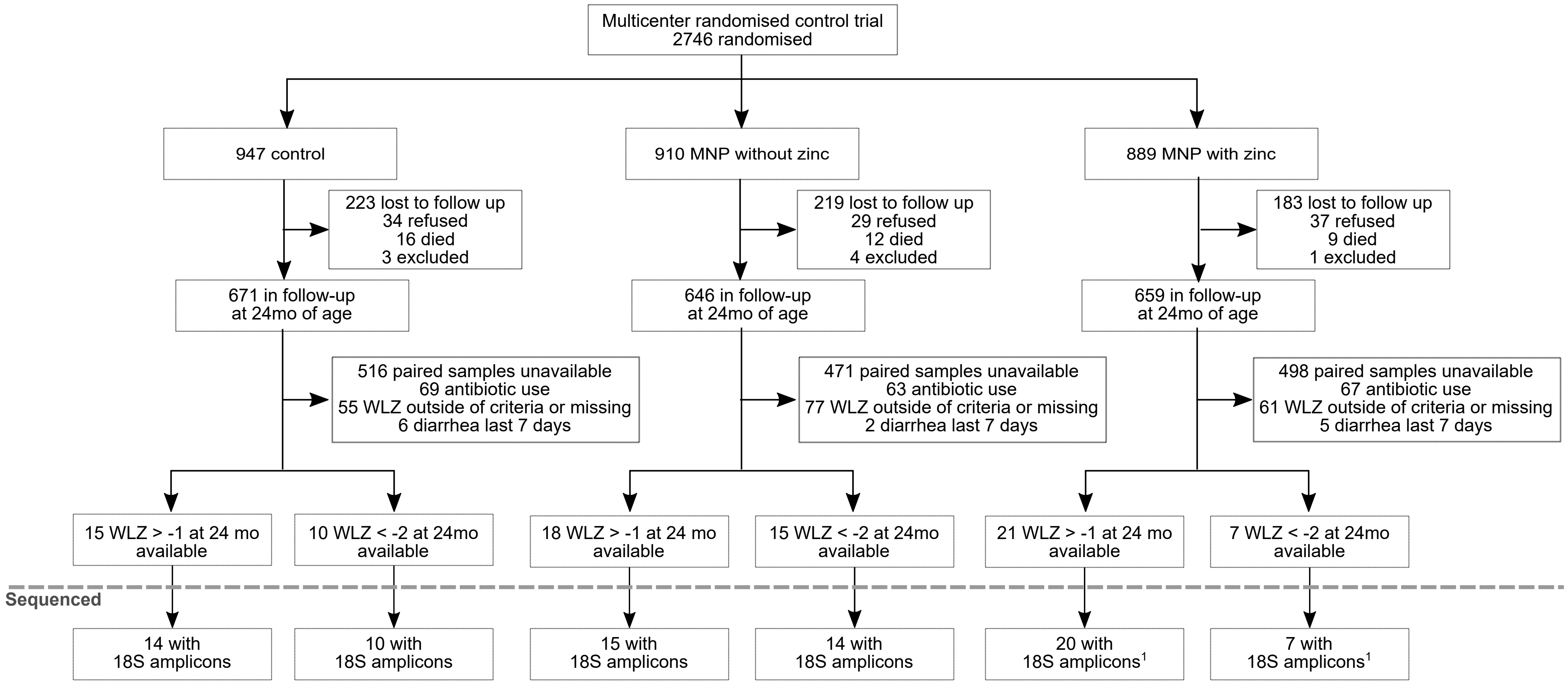

[1] Two subjects (one in the reference WLZ group and one undernourished) had, at 12 months, no diarrhea within 1 day of stool collection but reported diarrhea within 7 days prior.

Supplementary Figure 1. Selection of participants from micronutrient supplementation trial (ClinicalTrials.gov identifier NCT00705445) for profiling of stool microbiota.

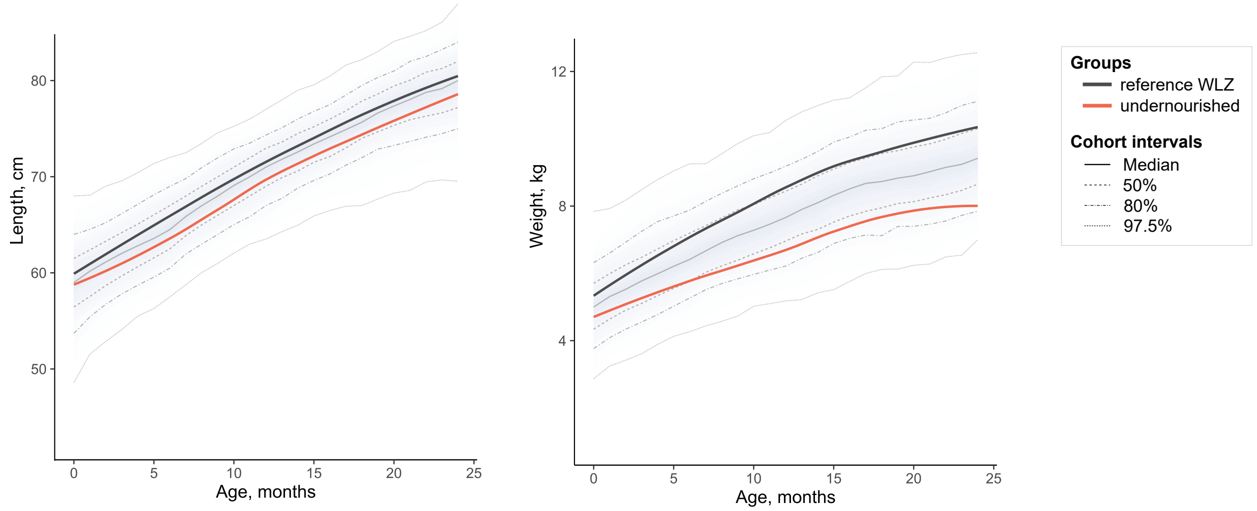

Supplementary Figure 2. Length (*left*) and weight (*right*) z-scores of children recruited into parent clinical trial NCT00705445 during the first 24 months of life. Median and quantile values are shown, with medians for participants profiled in the current study indicated by red (undernourished) and black (reference WLZ) lines.

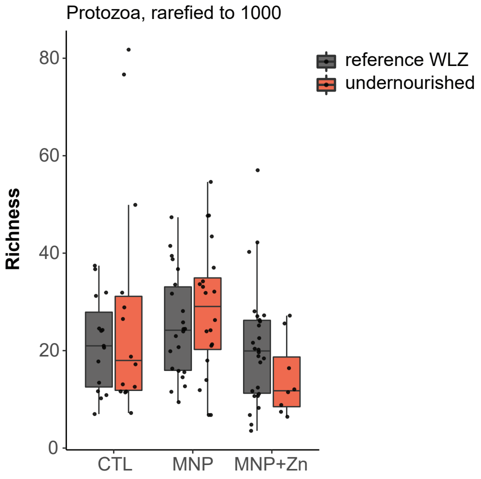

Supplementary Figure 3. Protozoan richness in stool samples, grouped by nutritional status and micronutrient supplementation. Samples (n=106) were rarefied to 1000 reads. Boxplots represent medians and interquartile ranges (IQR), and whiskers demarcate 1.5 x IQR.

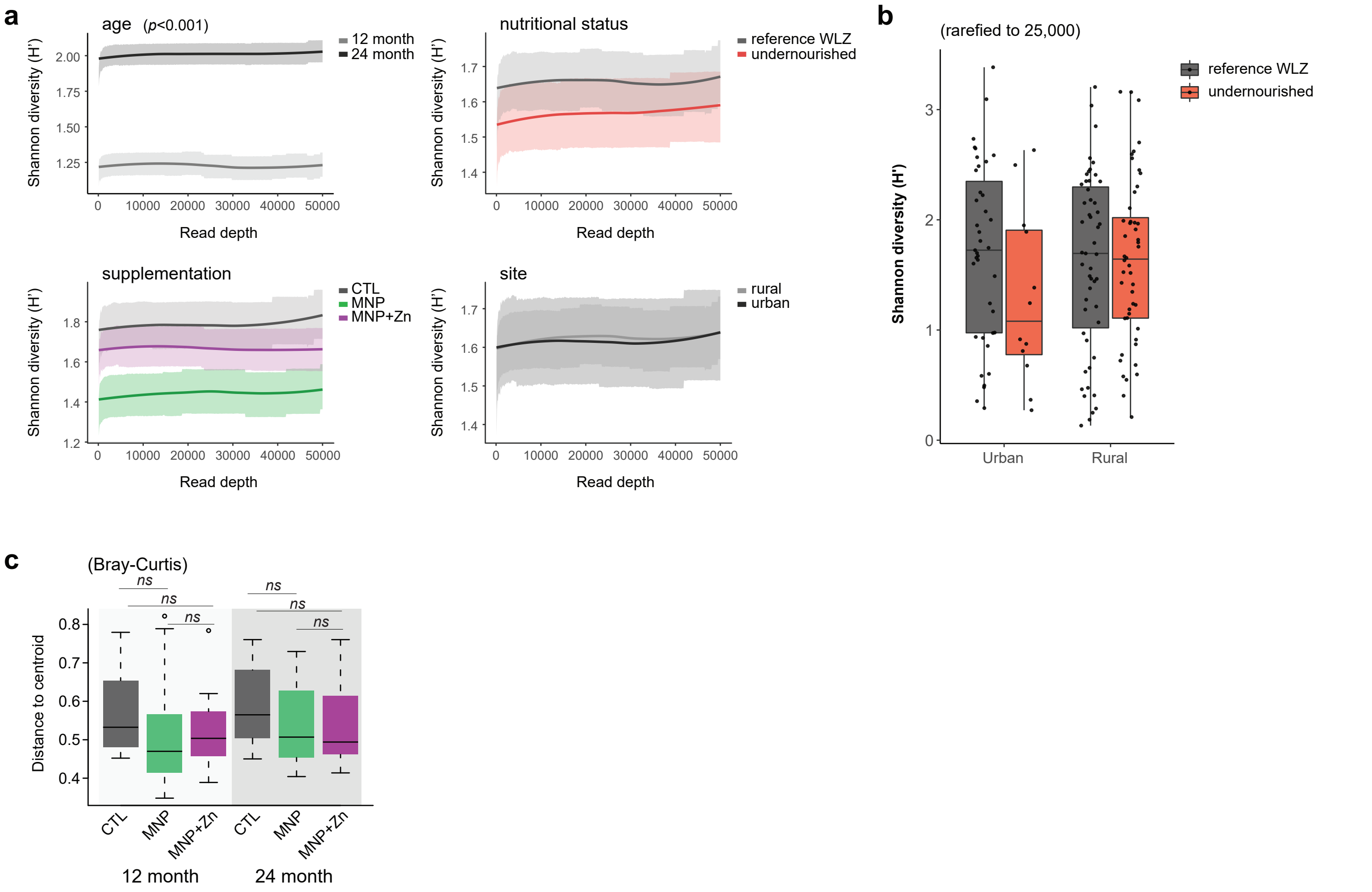

Supplementary Figure 4. Bacterial evenness and beta diversity. **a,** Rarefaction curves comparing species evenness (H′) by age group, micronutrient supplementation arm, nuritional status and site. Shaded regions represent standard error. A generalized linear model with stewise AIC-based model selection was implemented to identify significantly contributing variables at a read depth of 25,000, and *p* values were determined using the Wald test. **b,** Boxplots comparing bacterial evenness by nutritional status and site (n=150). **c,** Variance among Bray-Curtis dissimilarities, calculated as distance to the centroid, between samples (n=150) grouped by treatment arm and age. Significance was tested using two-way ANOVA with the Tukey HSD posthoc test. Boxplots in **b** and **c** represent medians and interquartile ranges (IQR), and whiskers demarcate 1.5 x IQR. ns, no significant difference.

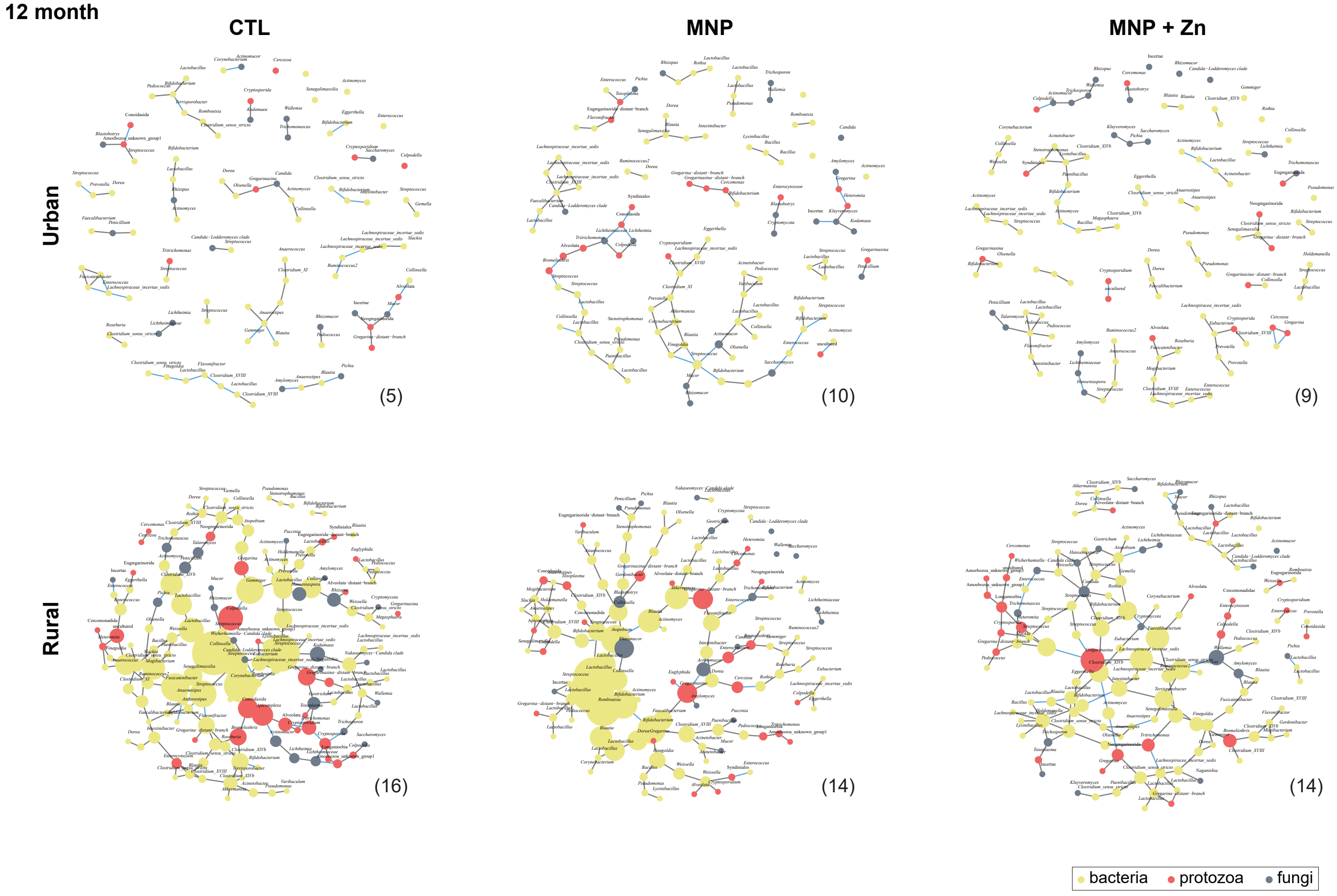

Supplementary Figure 5. Graphic representations of microbial networks representing predicted microbial interactions in 12 month old children, grouped by place of residence and micronutrient supplementation arm. Nodes represent bacterial OTUs (yellow) and protozoan and fungal genera (red and grey, respectively), scaled by betweenness centrality scores. Edges represent significant positive (grey) and negative (blue) correlations among microbiota. Taxa with no predicted interactions have been removed. Numbers of samples used to generate each network are indicated within brackets.

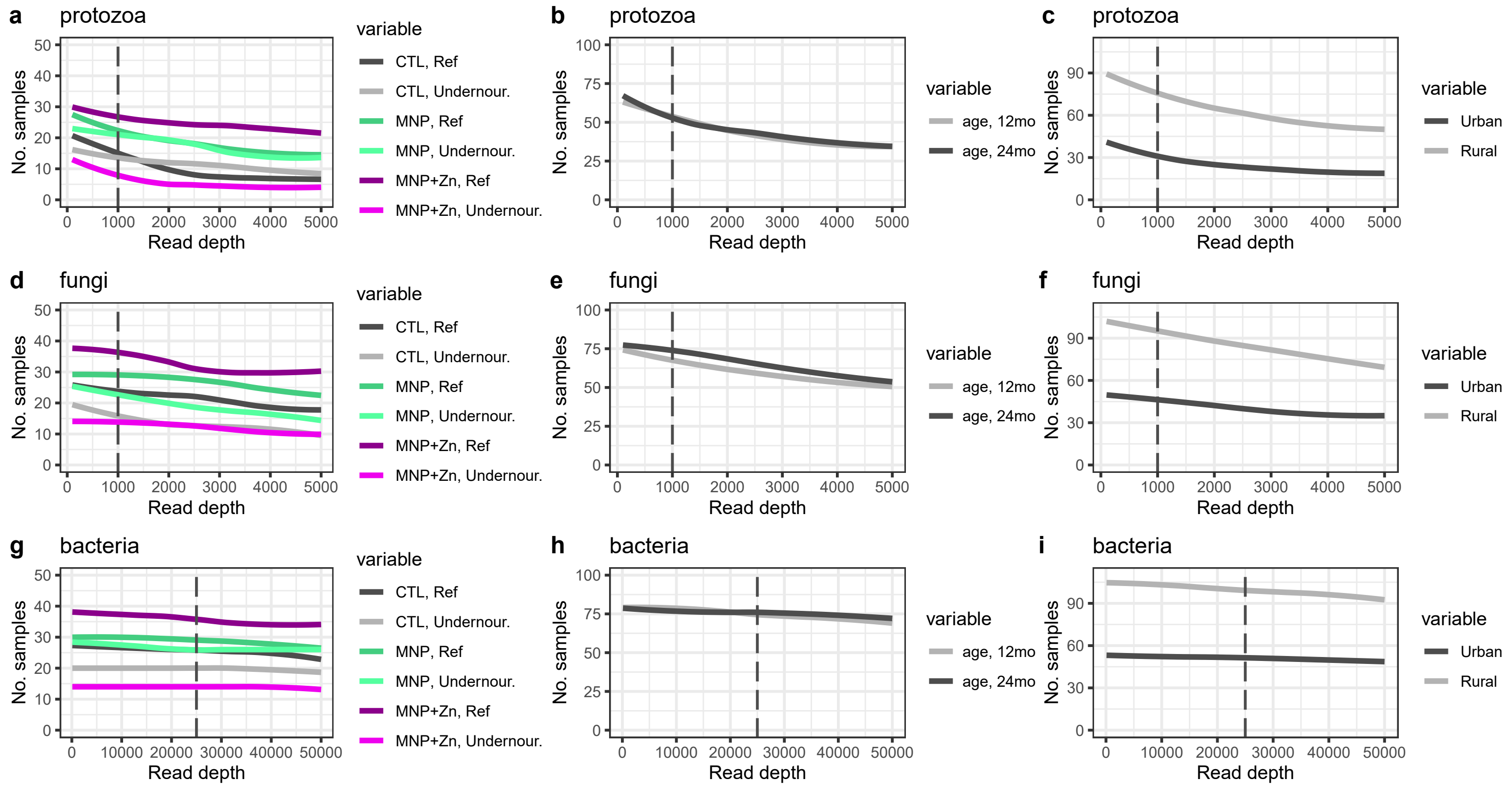

Supplementary Figure 6. Number of available samples per read depth of protozoan 18S sequences (**a-c**), fungal 18S sequences (**d-f**), and bacterial 16S sequences (**g-i**), in groups defined by supplementation and nutritional status (*left*), age (*middle*), or location of residence (*right*).
