# Supplementary material for: Micronutrient supplements can promote disruptive protozoan and fungal communities in the developing infant gut
Source: Nat Commun. 2021 Nov 18;12:6729. doi: 10.1038/s41467-021-27010-3 (PMC8602372; doi:10.1038/s41467-021-27010-3)
Supplement: Supplementary file 3 — Description of Additional Supplementary Files [file 41467_2021_27010_MOESM3_ESM.docx]

**Description of Additional Supplementary Files**

File Name: Supplementary Data 1
Description: 18S read and OTU summary

File Name: Supplementary Data 2

Description: 18S OTU table

File Name: Supplementary Data 3

Description: P-values for Figure 2d (eukaryotic carriage statistics in children grouped by supplementation arm) and Figure 4a (microbial interaction network density)

File Name: Supplementary Data 4

Description: 16S read and OTU summary

File Name: Supplementary Data 5

Description: 16S OTU table

File Name: Supplementary Data 6

Description: Partial Least Squares path modeling (PLS-PM)
