# Supplementary material for: Micronutrient supplements can promote disruptive protozoan and fungal communities in the developing infant gut
Source: Nat Commun. 2021 Nov 18;12:6729. doi: 10.1038/s41467-021-27010-3 (PMC8602372; doi:10.1038/s41467-021-27010-3)
Supplement: Supplementary file 10 — Reporting Summary [file 41467_2021_27010_MOESM10_ESM.pdf]

nature portfolio

Corresponding author(s): John Parkinson

Last updated by author(s): Oct 25, 2021

# Reporting Summary

Nature Portfolio wishes to improve the reproducibility of the work that we publish. This form provides structure for consistency and transparency in reporting. For further information on Nature Portfolio policies, see our Editorial Policies and the Editorial Policy Checklist.

## Statistics

For all statistical analyses, confirm that the following items are present in the figure legend, table legend, main text, or Methods section.

| n/a | Confirmed | |
|---|---|---|
| ☐ | ☒ | The exact sample size (*n*) for each experimental group/condition, given as a discrete number and unit of measurement |
| ☐ | ☒ | A statement on whether measurements were taken from distinct samples or whether the same sample was measured repeatedly |
| ☐ | ☒ | The statistical test(s) used AND whether they are one- or two-sided<br>*Only common tests should be described solely by name; describe more complex techniques in the Methods section.* |
| ☐ | ☒ | A description of all covariates tested |
| ☐ | ☒ | A description of any assumptions or corrections, such as tests of normality and adjustment for multiple comparisons |
| ☐ | ☒ | A full description of the statistical parameters including central tendency (e.g. means) or other basic estimates (e.g. regression coefficient) AND variation (e.g. standard deviation) or associated estimates of uncertainty (e.g. confidence intervals) |
| ☐ | ☒ | For null hypothesis testing, the test statistic (e.g. *F*, *t*, *r*) with confidence intervals, effect sizes, degrees of freedom and *P* value noted<br>*Give P values as exact values whenever suitable.* |
| ☒ | ☐ | For Bayesian analysis, information on the choice of priors and Markov chain Monte Carlo settings |
| ☐ | ☒ | For hierarchical and complex designs, identification of the appropriate level for tests and full reporting of outcomes |
| ☒ | ☐ | Estimates of effect sizes (e.g. Cohen's *d*, Pearson's *r*), indicating how they were calculated |

*Our web collection on statistics for biologists contains articles on many of the points above.*

## Software and code

Policy information about availability of computer code

| | |
|---|---|
| Data collection | Sequencing data was collected on the Illumina MiSeq with v3 chemistry (18S) or v2 chemistry (16S). |
| Data analysis | All sequence data pre-processing (quality filtering, merging and clustering) and taxonomic classification was carried out in CentOS Linux 7 using open source tools (USEARCH v11.0.667 and the UCLUST algorithm, Trimmomatic v0.36 and VSEARCH v2.10.4, BLAST 2.2.28 and SINA v1.2.11), as described in methods. Phylogenetic trees were constructed using FastTree 2.1 and visualized using the Iroki viewer. Analyses were performed in R 4.0.2 using freely available R packages: Phyloseq 1.20.0 and vegan 2.5-7 for microbial diversity analyses, DESeq2 1.22.2 for differential taxon abundance, SpiecEasi 1.1.0 and igraph 1.2.6 for microbial networks of associations, and the package plspm 0.4.9 for the development of an integrated model of cross-correlations among microbiota and demographic and clinical variables. The rstatix 0.7.0 package was used for Fisher's Exact or pairwise tests of eukaryotic carriage; the glm function in R and MASS 7.3-53.1 were used used to evaluate alpha diversity differences, and the adonis, capscale, envfit and betadisper functions in vegan were used to evaluate beta diversities.<br><br>R code for analyses is available in the GitHub repository ParkinsonLab/gut-eukaryotes-malnutrition-and-micronutrient-supplementation: https://doi.org/10.5281/zenodo.5606073 |

For manuscripts utilizing custom algorithms or software that are central to the research but not yet described in published literature, software must be made available to editors and reviewers. We strongly encourage code deposition in a community repository (e.g. GitHub). See the Nature Portfolio guidelines for submitting code & software for further information.

## Data

Policy information about availability of data

All manuscripts must include a data availability statement. This statement should provide the following information, where applicable:

- Accession codes, unique identifiers, or web links for publicly available datasets
- A description of any restrictions on data availability
- For clinical datasets or third party data, please ensure that the statement adheres to our policy

The 16S rRNA and 18S rRNA amplicon data generated in this study have been deposited in the NCBI Sequence Read Archive database under the accession code PRJNA717317 [https://www.ncbi.nlm.nih.gov/bioproject/?term=PRJNA717317]. The OTU count data generated in this study are provided in the Supplementary Information file.

The Ribosomal Database Project version 16 [https://sourceforge.net/projects/rdp-classifier/], SILVA v132 non-redundant reference database [https://www.arb-silva.de/download/archive/] and NCBI nucleotide database [https://www.ncbi.nlm.nih.gov/nucleotide/] used for taxonomic classification in this study are available under the provided weblinks.

# Field-specific reporting

Please select the one below that is the best fit for your research. If you are not sure, read the appropriate sections before making your selection.

☒ Life sciences ☐ Behavioural & social sciences ☐ Ecological, evolutionary & environmental sciences

For a reference copy of the document with all sections, see nature.com/documents/nr-reporting-summary-flat.pdf

# Life sciences study design

All studies must disclose on these points even when the disclosure is negative.

| | |
|---|---|
| Sample size | Eighty participants were selected for profiling of intestinal eukaryotic and bacterial communities at 12 and 24 months of age (160 samples in total). Limited sample availability precluded a balanced design with respect to nutritional status and micronutrient supplementation. |
| Data exclusions | Pre-determined exclusion criteria included not having paired samples, not being within weight-for-height criteria, having a record of antibiotic administration within one month of sample collection, and having reported diarrhea seven days prior or on the day of stool collection. |
| Replication | As a retrospective analysis of microbiota (including eukaryotes) in children from a cluster randomized controlled trial (cRCT) performed in low-resource settings, there exist to our knowledge no comparable cohort to serve as replicate in this study. We hope to engage with similar research effort and cohort to replicate/validate results in an independent cohort. All codes are available for reproducibility. |
| Randomization | The parent trial was cluster randomised: 256 clusters were identified and randomly assigned within urban and rural strata to receive non-supplemented, micronutrient powder without zinc, or micronutrient powder with zinc. Sample and participant selection for the substudy was based on sample availability, weight-for-length criteria, and no record of antibiotic treatment or diarrhea prior to stool sample collection. The 'reference' group was chosen to represent the healthiest possible comparator group based on fewest weight-for-length z-scores below -1 reported between 6 and 24 months of age. |
| Blinding | Micronutrient powder sachets were identical but colour coded; and investigators were masked to composition of powders until trial completion. Parents knew whether their child was receiving supplementation, but did not know whether the powder contained zinc. The retrospective microbiome study was not blinded to group allocation during data analysis. Pre-selected grouping variables (nutritional status, age, locality and micronutrient supplementation) were tested for association with predetermined metrics (microbial diversity and differential taxon abundance). |

# Reporting for specific materials, systems and methods

We require information from authors about some types of materials, experimental systems and methods used in many studies. Here, indicate whether each material, system or method listed is relevant to your study. If you are not sure if a list item applies to your research, read the appropriate section before selecting a response.

## Materials & experimental systems

| n/a | Involved in the study |
| --- | --- |
| ☒ | ☐ Antibodies |
| ☒ | ☐ Eukaryotic cell lines |
| ☒ | ☐ Palaeontology and archaeology |
| ☒ | ☐ Animals and other organisms |
| ☐ | ☒ Human research participants |
| ☐ | ☒ Clinical data |
| ☒ | ☐ Dual use research of concern |

## Methods

| n/a | Involved in the study |
| --- | --- |
| ☒ | ☐ ChIP-seq |
| ☒ | ☐ Flow cytometry |
| ☒ | ☐ MRI-based neuroimaging |

# Human research participants

Policy information about studies involving human research participants

| | |
| --- | --- |
| Population characteristics | Eighty participants were subselected from the parent trial for profiling of microbiota in previously bioarchived stool samples. Of these, 44 (55%) were female, 31 were undernourished with weight-for-length *z*-scores (WLZ) <-2, and 49 were in the reference WLZ comparator group (WLZ >-1). Numbers of participants by micronutrient supplementation arm are as follows: 24 (30.0%) unsupplemented controls, 29 (36.2%) supplemented with micronutrient powders (MNPs) and 27 (33.8%) supplemented with MNPs with zinc. The substudy profiled stool samples from each participant at 12 and 24 months of age. |
| Recruitment | Recruitment for the parent cRCT is described in Soofi et al. 2013. The Lancet. 382:29-40. doi: 10.1016/S0140-6736(13)60437-7 |
| Ethics oversight | All clinical data and stool samples used for analysis were collected in a previously conducted cRCT (NCT00705445) that was approved by the Ethics Review Committee (ERC) of AKU (752-Peds/ERC-07). All participants enrolled in the parent cRCT (NCT00705445) provided written informed consent for the routine collection of stool specimens for analysis of their intestinal microbial communities. Ethical approval for the retrospective analysis of clinical data and stored stool samples was granted by the research ethics board (REB) at SickKids, Toronto (REB No. 1000054244), the ERC at AKU, Karachi, Pakistan (4840-Ped-ERC-17), and the National Bioethics Committee (NBC) Pakistan (4-87/NBC-277/17/1191). A waiver of consent for this retrospective analysis was granted by each of the above-mentioned regulatory boards. |

Note that full information on the approval of the study protocol must also be provided in the manuscript.

# Clinical data

Policy information about clinical studies

All manuscripts should comply with the ICMJE guidelines for publication of clinical research and a completed CONSORT checklist must be included with all submissions.

| | |
| --- | --- |
| Clinical trial registration | Samples were obtained from a previous cRCT (ClinicalTrials.gov identifier NCT00705445). |
| Study protocol | The study protocol of the parent trial is described in Soofi et al. 2013. The Lancet. 382:29-40. doi: 10.1016/S0140-6736(13)60437-7, and Ariff et al. 2014. J Nutr. 144:20-26. doi: 10.3945/jn.113.178715 |
| Data collection | Data collection for the original cRCT is described in Soofi et al. 2013. The Lancet. 382:29-40. doi: 10.1016/S0140-6736(13)60437-7 |
| Outcomes | Profile of eukaryotic and bacterial communities using 18S and 16S rRNA amplicon sequencing, and association of microbial diversities and compositions with 1) nutritional status, 2) micronutrient supplementation, 3) geographic locality and 4) age. |



March 2021
